# Hybridization of poly(rI) with poly(rC) adsorbed to the carbon nanotube surface

**DOI:** 10.1186/1556-276X-9-157

**Published:** 2014-04-01

**Authors:** Maksym V Karachevtsev, Galyna O Gladchenko, Victor S Leontiev, Victor A Karachevtsev

**Affiliations:** 1 B.I. Verkin Institute for Low Temperature Physics and Engineering, National Academy of Sciences of Ukraine, Kharkov 61103, Ukraine

**Keywords:** Carbon nanotubes, Homopolynucleotide, Poly(rC), Poly(rI), Hybridization, Absorption spectroscopy, Molecular dynamics

## Abstract

Hybridization of homopolynucleotide poly(rC) adsorbed to the carbon nanotube surface with poly(rI) free in solution has been studied by absorption spectroscopy and molecular dynamics method. It was found that hybridization on the nanotube surface has a slow kinetics, the behavior of which differs essentially from fast hybridization of free polymers. The duplex obtained is characterized with the reduced thermostability and a lower hyperchromic coefficient than it was observed when the duplex was formed in the absence of the nanotube. These features point to the imperfectness in the structure of the duplex hybridized on the nanotube surface. Computer simulation showed that the strong interaction of nitrogen bases with the nanotube surface weakens significantly hybridization of two complementary oligomers, as the surface prevents the necessary conformational mobility of the polymer to be hybridized.

## Background

Detection of DNA sequences through hybridization between two complementary single strands is a basic method that is very often exploited at the DNA biosensor development [1]. Now new opportunities have appeared in this route due to synthesis of new nanomaterials which are intensively applied as the scaffold, transducer, or sensitive detectors. In particular, carbon nanotubes have attracted keen interest of biosensor researchers [2]. It was found that single-stranded nucleic acid (ssDNA) binds to the single-walled carbon nanotube (SWNT), forming a stable soluble hybrid in water [3]. In spite of the essential difference in structures of nanotubes and the biopolymer, ssDNA wraps tightly around the nanotube in water when hydrophobic nitrogen bases are adsorbed onto the nanotube surface via π-π stacking, while the hydrophilic sugar-phosphate backbone is pointed towards water [3,4].

The hybridization of nucleic acids on SWNT is extensively investigated [5-22], having in sight the development of DNA-hybridized biosensors on the base of nanotubes. Nevertheless, in spite of 10-year investigations in this field, some questions arise upon the study of DNA hybridization on the nanotube especially when the probe polymer was adsorbed to the tube surface directly. One of the keen questions is the effect of DNA interaction with the tube surface on the polymer hybridization. Effective detection of hybridization of two complementary DNA strands on the nanotube surface was demonstrated in [5-7]; however, in other measurements [12,14,17], it was indicated that SWNT hampers effective hybridization of two polymers because of the strong interaction with the nanotube surface, which prevents the necessary conformational mobility of the polymer to be hybridized. Some researchers suppose that the double-stranded DNA (dsDNA) is desorbed from the sidewall of SWNT after hybridization [14,18-22]. Thus, up to now, the full picture of the biopolymer hybridization on SWNT surface is still unclear, and in some cases, the conclusions are controversial. To clarify this ambiguity, an additional study is required. In this work, we focus our research on the hybridization of polyribocytidylic acid (poly(rC)) adsorbed to the carbon nanotube surface with polyriboinosinic acid (poly(rI)) free in solution. The choice of homopolynucleotides for studying the nucleic acid hybridization on the nanotube was based on extensive experimental information on these well-studied model systems, with the desire to avoid ambiguity in the result interpretation, appearing sometimes upon studying the heterogeneous base sequence. Homopolynucleotides are often used to study biopolymer adsorption on the nanotube; in particular, these polymers reveal various affinities to the carbon surface, depending on their rigidity [23]. Moreover, homopolynucleotides are the most suitable systems to study association of complementary strands since this bimolecular second-order reaction occurs quite rapidly [24]. The substantial argument is the relatively low costs of homopolynucleotides as often this factor becomes a stumbling block in the way of practical application.

There is also another significant problem which has encouraged the choice of these polymers. Double-stranded poly(rI)∙poly(rC) plays an important biological role in the activation of the human innate immune system and adaptive immune responses, and triggers directly apoptosis in cancer cells [25,26]. On other hand, it was also shown that a SWNT-modified DNA probe has increased self-delivery capability and intracellular biostability when compared to free DNA probes [27]. In addition, as carbon nanotubes are an effective drug delivery scaffold, their combination with poly(rI)∙poly(rC) may find new applications in clinical practice.

To study the hybridization of poly(rI) with poly(rC) on the carbon nanotubes, in this work, we try to combine experiments (UV absorption spectroscopy) and computer modeling (molecular dynamics method).

## Methods

### Materials

Potassium salts of poly(rC), poly(rI), and duplex poly(rI)∙poly(rC) (Sigma-Aldrich, St. Louis, MO, USA) were used as received. The polymers were dissolved in 0.01 M Na^+^ cacodylate buffer (pH 7) (Serva, Heidelberg, Germany) with 0.06 M NaCl, and 0.2 mM Na_2_EDTA (Sigma). For the buffer preparation, the ultrapurified water with resistivity of 18 MΩ∙cm^−1^ obtained from Millipore Super-Q system (Millipore Co., Billerica, MA, USA) was used. The concentration of polynucleotide phosphates ([P]) was determined spectrophotometrically using the molar extinction coefficients: poly(rC), *ϵ*_268_ = 6,300 M^−1^∙cm^−1^[28,29]; poly(rI), *ϵ*_248_ = 10,100 M^−1^∙cm^−1^[30]; and poly(rI)∙poly(rC), *ϵ*_260_ = 4,800 M^−1^∙cm^−1^[31]. Purified HiPCO® single-walled carbon nanotubes were purchased from Unidym (Sunnyvale, CA, USA).

For preparing poly(rC):SWNT conjugates, carbon nanotubes were mixed with an aqueous solution of poly(rC) at 1.2:1 mass ratio. The initial concentration of SWNTs was ≈ 200 mg/l. The samples were ultrasonicated for 40 min (1 W, 44 kHz) in an ice-water bath by using a USDN-2 T probe sonicator (Selmi Inc., Sumy, Ukraine). After 40 min of sonication, the RNA solution contains fragments, the lengths of which were within 100 to 300 nucleotides. Influence of the ultrasound exposure time on the length of DNA fragments was investigated by agarose gel-electrophoresis according to the procedure described in [32]. After sonication, the suspension was centrifuged at 70,000 *g* for 1 h; then, the supernatant was decanted and dialyzed (dialysis tubing with a molecular weight cutoff of 13 to 14 kDa) against the buffer solution for 36 h to remove free polynucleotides which did not adsorb to SWNTs.

In the next step, poly(rC):SWNT conjugates were hybridized with the complementary poly(rI) in buffer solution by mixing equimolar amounts ((1 ÷ 6) × 10^−5^ M [P]) of fragmented polymers in buffer with those adsorbed to the nanotube surface. For comparison, under identical conditions (including the preliminary ultrasound treatment for 40 min), the hybridization of free polymers was carried out, too. We selected the temperature equal to 20°C for poly(rI) and poly(rC) hybridization on the basis of the fact that the maximum rate of this process occurs at a temperature of about 25°С lower than the melting temperature (*T*_m_) for the duplex [33]. The temperature of the helix-coil transition in poly(rI)∙poly(rC) has been determined earlier [34] as *T*_m_ ≈ 57°C. Also, it was shown that the melting temperature of the duplex hybridized onto the nanotube decreases in comparison with that of the free one [17]. As the bell-shaped curve relating hybridization rate and temperature is broad, with a rather flat maximum from about 16°C to 32°C below *T*_m_, the temperature equal to 20°C is the optimal value.

### Absorption spectroscopy

Differential UV-visible absorption spectroscopy was used for analysis of structural changes in polynucleotides at their interaction with carbon nanotubes. Absorbance measurements and melting experiments were carried out on spectrophotometer Specord M40 (Carl Zeiss, Jena, Germany) using 1-cm path length quartz cuvettes. Temperature dependences of the increase in the optical density (Δ*A*(*T*)) of polynucleotides were measured by means of a two-cuvette differential arrangement - one cuvette in each channel of the spectrophotometer. Both cuvettes contained the identical concentration of polynucleotide solutions or of polynucleotide:SWNT suspensions. The reference cell was thermostated within 20 ± 0.5°C; the working one was heated at the rate of 0.25°C/min. The buffer polymer solution and suspension with nanotubes were vacuum-degassed prior to melting experiments to minimize the bubble formation at high temperatures. Melting curves of poly(rI)∙poly(rC) (free or bonded with nanotubes) were measured at *λ* = 248 nm as *h*(*T*) = Δ*A*(*T*)/*A*_0_ where *A*_0_ is the optical absorption of the folded (initial) polymer, Δ*A* is determined as Δ*A* = (*A* − *A*_0_), and *h*(*T*) is the hyperchromic coefficient. Hybridization of poly(rI) with poly(rC) in solution or on nanotubes was monitored through the UV optical absorption decrease (at *λ*_max_ = 248 nm) which is usually observed after the formation of the double-stranded helix (the so-called hypochromic effect which is opposite to the hyperchromic one).

### Molecular dynamics simulation

The formation of hybrid r(C)_25_ with SWNT was simulated by the molecular dynamics method. For this purpose, the program package NAMD [35] was employed with Charmm27 force field parameter set [36]. Before starting the simulation, the oligonucleotide (in A-conformation) was located near the nanotube surface. Twenty-five Na^+^ ions were added to the system for neutralization of the charge on the sugar-phosphate backbone. SWNT was selected as a zigzag (16,0) nanotube. Its length and diameter were 11.0 and 1.122 nm, respectively. SWNT atoms were uncharged. For modeling, periodical boundary conditions were provided (box's size 50 Å × 140 Å × 65 Å). Hybrid was embedded in water (more than 14,000 H_2_O molecules). The system was minimized during 1,000 steps (with 1-fs time step) and then modeled during 50 ns (time step was also 1 fs). The first 2 ns of simulation time was considered as an equilibration step; this time was not taken into account for data analysis. In our simulations, NPT ensemble was used. Isobaric-isothermal ensemble (NPT) is characterized by a fixed number of atoms, *N*, a fixed pressure, *P*, and a fixed temperature, *T*. The temperatures and pressures in the periodic boxes were 343 K and 1 atm, respectively. The temperature of the simulated system was selected in accordance with our earlier results [37] indicating that the temperature growth increases the rate of achieving the energetically more favored conformation of oligomer on the nanotube mainly because of the destruction of nitrogen base self-stacking. As a result, this makes easier the process of the oligomer wrapping around the nanotube. The temperature rise in the moderate range increases the hybridization rate, too [38].

After 50 ns modeling, free r(I)_10_ (in A-conformation) was added to the system. Ten Na^+^ ions were added to the system for neutralization of the charge on the r(I)_10_. Temperature, pressure, and periodic boundary conditions were the same as in the case of the previous modeling. Interaction energies were calculated by the NAMD Energy Plugin (version 1.3) which was implemented in the VMD program package [39].

## Results and discussion

### Spectroscopic investigation of poly(rI) hybridization with poly(rC)

**Figure 1:**
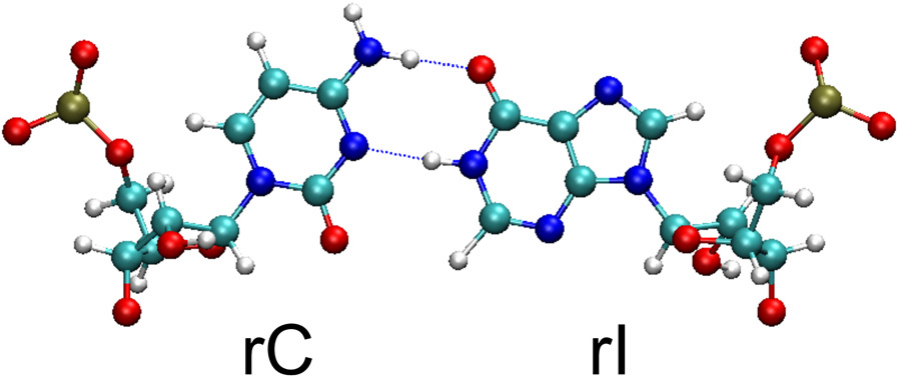
**Hybridized rI-rC structure with Watson-Crick base pairing.** Blue balls - N, green balls - C, gray balls - H, red balls - O, and deep-yellow balls - P.

At first, we have studied the hybridization of fragmented poly(rI) and poly(rC) in aqueous solution to compare this process with the polymer hybridization on the nanotube surface. At neutral pH and middle ionic strengths (0.07 M Na^+^) of solution, poly(rC) forms with poly(rI) the double-stranded helix in which Watson-Crick base pairs have two hydrogen bonds between hypoxanthine of one strand and cytosine of the opposite strand (Figure 1) [31].

**Figure 2:**
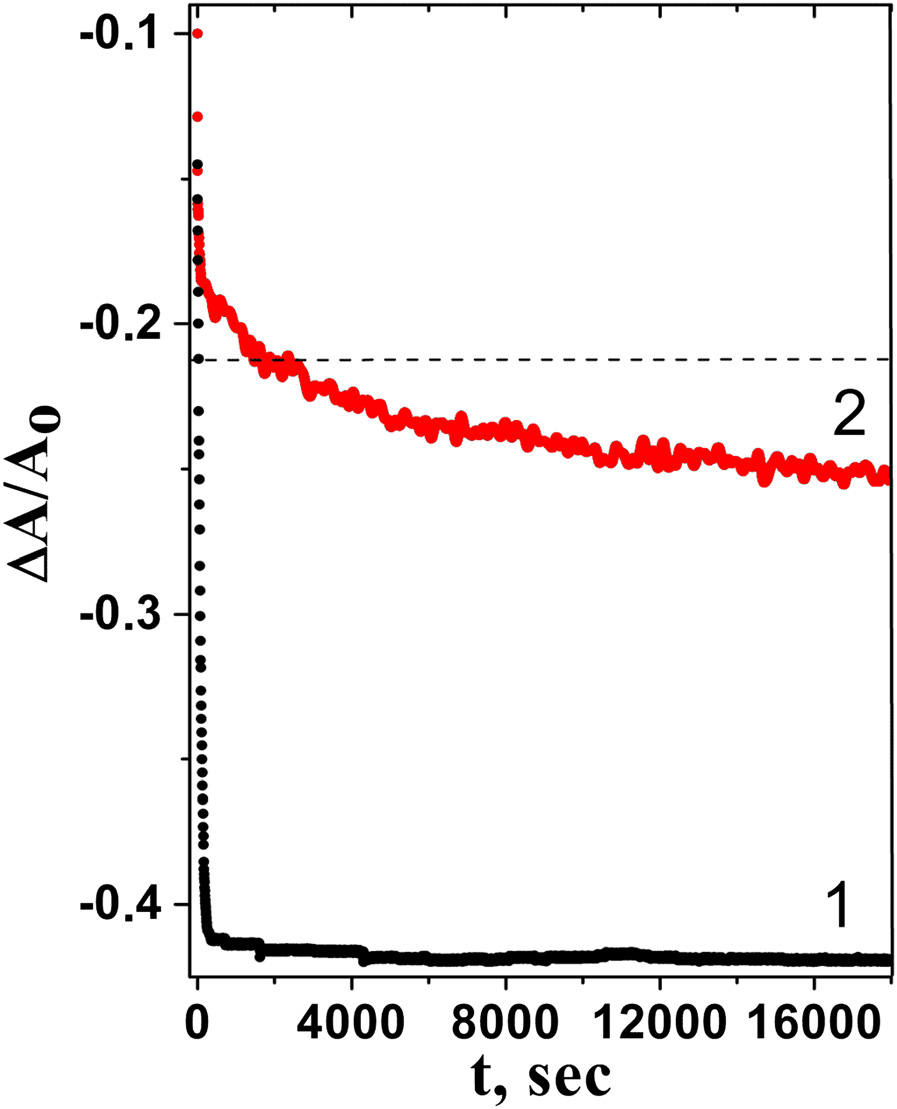
**Time dependences of absorption hypochromism (*λ*** **= 248 nm) observed at mixing.** 1, water solutions of poly(rC) and poly(rI); 2, poly(rC)^NT^ suspension and solution of poly(rI). Kinetics was measured at 20°C. The dashed line corresponds to the formation of 50% of the base pairs.

Figure 2 (curve 1) shows the time dependence of the hypochromic coefficient for the duplex of two homopolymers upon its formation, starting from the mixing of poly(rI) and poly(rC) solutions. Note that the decrease of this coefficient indicates the appearance of double-stranded (ds-) poly(rI)∙poly(rC) in aqueous solution. As follows from this dependence, poly(rI)∙poly(rC) formation in solution is characterized with two stages: very fast kinetics in the initial time (nucleation and growth of helical regions, according to [40]) and very slow final phase. So, immediately after mixing of two polymer solutions (during approximately 30 s), about 50% of the base pairs (from all possible pairs) are formed, and then within the next 3 min, their number reaches 93% (Figure 2, curve 1). The final phase is characterized with a slow rate of polymer hybridization; so for 5 h, the number of pairs increases only by 5%. In this time period, the relaxation processes in irregular parts of the polymer like the loop occur [40,41]. It should be noted that, within 24 h after mixing of initial solutions, the hypochromic coefficient reaches its maximal value (*h*_max_ = 0.425). The fraction of bases in the double-stranded form (the degree of hybridization) can be obtained by using the simple ratio (*h*_t_/*h*_max_) in which the hypochromic coefficient at any time (*h*_t_) is compared with its maximal value.

**Figure 3:**
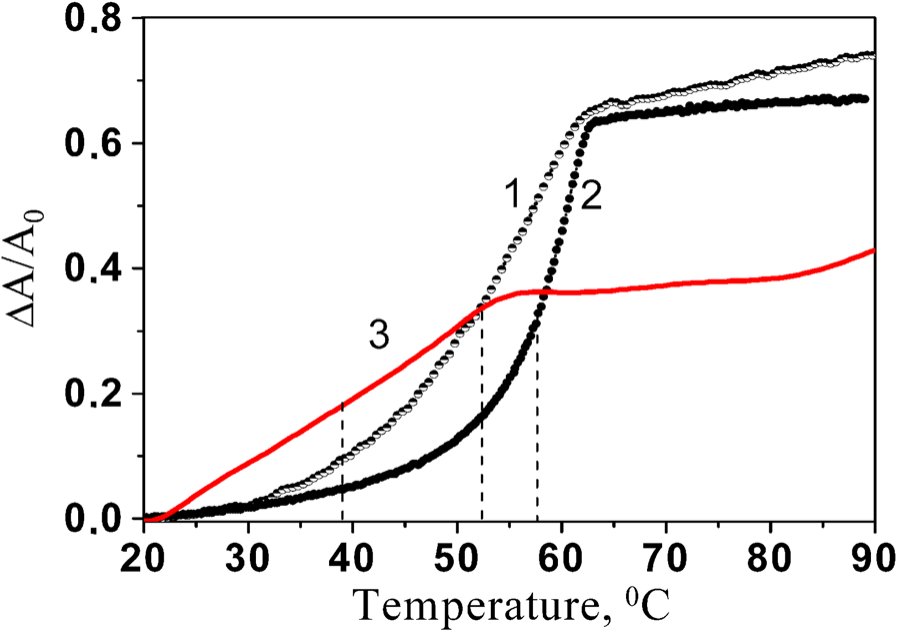
**Melting curves measured at *λ*** **= 248 nm.** 1, poly(rI)∙poly(rC) hybridized in buffer solution; 2, initial double-stranded poly(rI)∙poly(rC) (Sigma); 3, poly(rI)∙рoly(rC)^NT^ formed after 24 h of hybridization. The dashed lines indicate the positions of the melting temperatures of the corresponding curves.

To confirm the formation of the poly(rI)∙рoly(rC) duplex under these experimental conditions, we melted this polymer obtained after the hybridization (Figure 3, curve 1). As a result, we observed an S-like temperature dependence of light absorption (Figure 3, curve 1) that is evidence of the helix-coil transition in ds-RNA obtained due to hybridization. The melting temperature (*T*_m_) of the hybridized poly(rI)∙poly(rC) was found at 52.5°C. *T*_m_ is a standard measure of the solution thermodynamic stability of the duplex of nucleic acids, which is defined as a temperature at which the hypochromic coefficient reaches half of its value. This temperature also indicates the coexistence of half of the polymer in the duplex and in single strands.

We compared also the melting curve of hybridized poly(rI)∙poly(rC) with the curve obtained for the initial duplex poly(rI)∙рoly(rC) (Figure 3, curve 2). It turned out that the melting curve of the last polymer is shifted to a higher temperature. *T*_m_ value for this polymer is 57.7°C. It means that the thermostability of hybridized poly(rI)∙poly(rC) is reduced in comparison with that of the initial duplex poly(rI)∙poly(rC), while hyperchromic coefficients taken for the both curves almost coincide. In our opinion, the main reason of the thermostability decrease of the hybridized polymer is conditioned with polymer fragmentation caused by ultrasonication. As a result, poly(rI) and poly(rC) are shortened to fragments with 100 to 300 nucleotides in length. Due to some distribution in the length, the duplexes obtained after hybridization are characterized with the presence of dangling ends composed of single strands. This state manifests itself in the melting curve [42], the shape of which acquires the slight slope in the low-temperature part and the broadening of helix → coil transition in comparison with the initial duplex (18°C vs 8°C). Note that there is a difference in absolute values of hypochromic (Figure 2, curve 1) and hyperchromic (Figure 3, curve 1) coefficients. This difference disappears after taking into account the contribution of the hyperchromic effect of the ordered poly(rC) in the total hyperchromic coefficient at heating [43]. The similar contribution of poly(rI) in this melting curve is insignificant because this polymer is characterized with base disordering even at room temperature [23].

### Hybridization of free poly(rI) with poly(rC) adsorbed to SWNT

Hybridization kinetics of poly(rI) with poly(rC) adsorbed to the nanotube surface (poly(rC)^NT^) is different from that observed for free polymers by a smaller value of the hypochromic coefficient, although shapes of time dependences are similar (Figure 2, curve 2). In the fast stage of kinetics, about 40% of base pairs are formed after the first 80 s. Comparing the times taken for the formation of 50% of base pairs (*t*_1/2_), we found a slowdown of hybridization kinetics of polymers on the nanotube of 80 times (*t*_1/2_ ≈ 40 min), compared to the hybridization kinetics of free polymers in solution for which *t*_1/2_ was 30 s. Then, the kinetic of this process becomes linear with time, so that for approximately 4.5 h, the number of base pairs increases by 10% and runs up to 60% that corresponds to the hypochromic coefficient of 0.25. It should be noted that by this time, the hybridization process slows down, and for the following 19 h, the increase in the number of base pairs was no more than 22%. For 24 h, the total part of hybridized pairs was about 82% that resulted from a value of the hypochromic coefficient equal to 0.35. Similar time dependence was observed for kinetics of dsDNA formed with 20-bases linear DNAs on SWNT [18]. Slowing down of kinetics in the final stage is due to the steric constraints that inhibit the formation of hydrogen-bonded cytosine-hypoxanthine pairs and block zippering process [44,45]. Similar behavior of hybridization kinetics of two complementary DNAs (or RNAs) on the nanotube was observed earlier [6,17].

The melting curve of poly(rI) · рoly(rC)^NT^ after 24-h hybridization is shown in Figure 3 (curve 3). It should be noted that upon poly(rC) adsorption onto the nanotube, the self-stacking of bases is lost [23], and therefore, the contribution of poly(rC) hyperchromicity is practically absent, and curve 3 represents mainly destruction of poly(rI) · рoly(rC)^NT^ double-stranded parts. Comparing melting curves 1 and 3 (Figure 3), we can see that the duplex hybridized on the tube is characterized with a lower thermal stability (*Т*_m_ = 38°C). Also, the form of the melting curve 3 changes essentially (the curve becomes more flat), the temperature interval of the transition increases (Δ*T* ≈ 27°С), and the hyperchromic coefficient lowers (*h* ≈ 0.37). Similar behavior was observed for hybridization of poly(rU) with poly(rA) adsorbed to SWNT [17]. It should be noted that upon heating, some part of poly(rC) and, in a smaller extent, of poly(rI) bases can unstack from the surface. This process can contribute to the hyperchromic effect [4]. Lower thermal stability was observed for decamers hybridized on the individual carbon nanotube [15] and for DNA linked to gold nanoparticles [46]. Most likely, the decrease of the thermal stability of the double-stranded polymer hybridized on the solid surfaces or nanoparticles is a general observation, which occurs due to interactions between the polymers and the surface.

A lower value of the hyperchromic coefficient and a broad interval of the helix-coil transition which starts actually from room temperatures point to the heterogeneity of the double-helical structure hybridized on the carbon nanotube surface. DNA melting at room temperature indicates the presence of very short unstable sections in the duplex structure. Obviously, such a heterogeneity in the poly(rI)∙рoly(rC)^NT^ structure is a result of the strong polymer interaction with the nanotube surface, which makes difficult the successive hybridization along the whole polymer length. The small value of the hyperchromic coefficient indicates that a part of the bases does not take part in hybridization and other ones form defective base pairs distorted with the curvature of the nanotube surface on which hybridized pairs do not reach the conformation with the optimal energy. It is likely that in this case, only one H-bond is created between nitrogen bases [17]. Of course, the presence of only one H-bond does not decrease directly the stacking and hyperchromic coefficient of the duplex. However, weak base pairing because of the missing second H-bond may result in larger twisting of bases in the pair and, in turn, in the decrease of stacking between the neighbors along chain bases.

### Simulation of hybridization between r(I)_10_ and r(C)_25_ adsorbed to SWNT (r(C)_25_^NT^)

**Figure 4:**
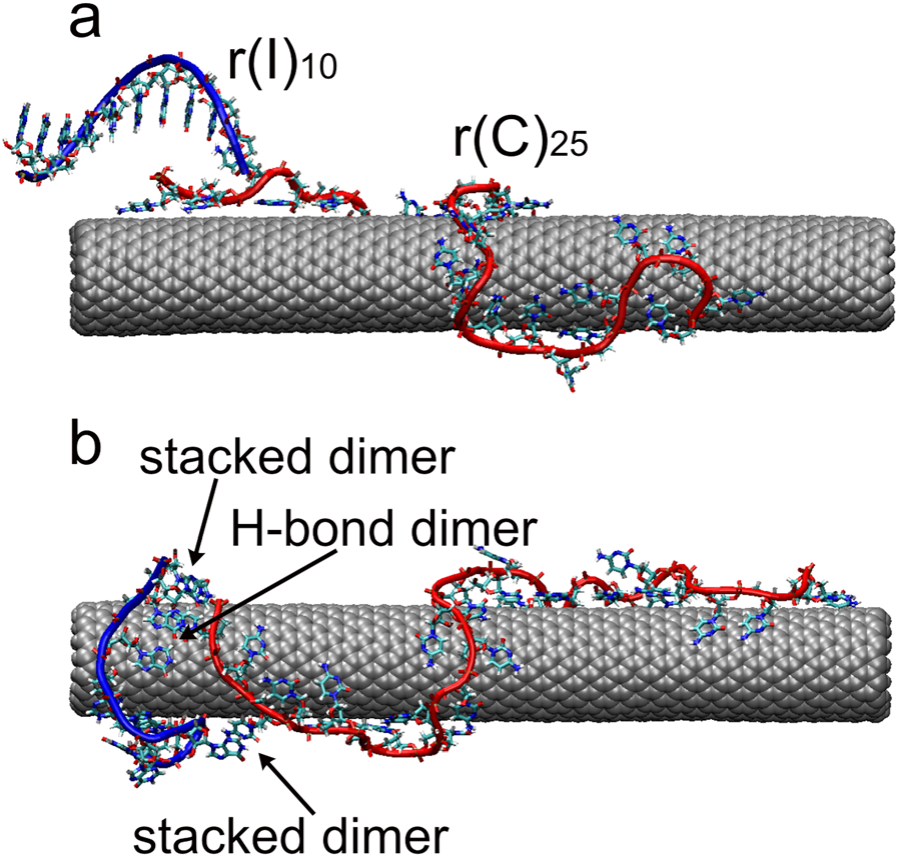
**Snapshot of r(I)**_**10 **_**and r(C)**_**25 **_**adsorbed to SWNT (16,0). (a)** In the initial simulation step and **(b)** after 50-ns simulation. Water molecules and Na^+^ counterions were removed for better visualization. The sugar-phosphate backbone of r(C)_25_ and r(I)_10_ is shown by red and blue strip, respectively.

We have studied the hybridization process of two complementary homooligonucleotides on the nanotube surface, employing the molecular dynamics method. For hybridization, two complementary homooligonucleotides, r(C)_25_ and r(I)_10_, were selected. At the beginning of simulation, r(C)_25_ was placed near the zigzag nanotube (16,0) and its adsorption was modeled for 50 ns. As it was mentioned above, these two oligomers differ from one another with the degree of base ordering, and as a result, they have different rigidities of the polymeric chains [23]. Earlier simulation of such oligomer adsorption onto the nanotube surface showed that the rigid oligomer r(C)_25_ turns spontaneously around the tube with a big pitch (Figure 4), and the flexible r(I)_25_ is placed more compactly on the tube, forming a stable loop that is apart from the nanotube [23]. The first oligomer has a higher energy of binding with the tube than the flexible one (325 kcal/mol vs 250 kcal/mol). After 50-ns modeling of spontaneous adsorption of r(C)_25_ onto the nanotube (at 343 K), 19 cytosines (from 25) were stacked with the nanotube surface.

**Figure 5:**
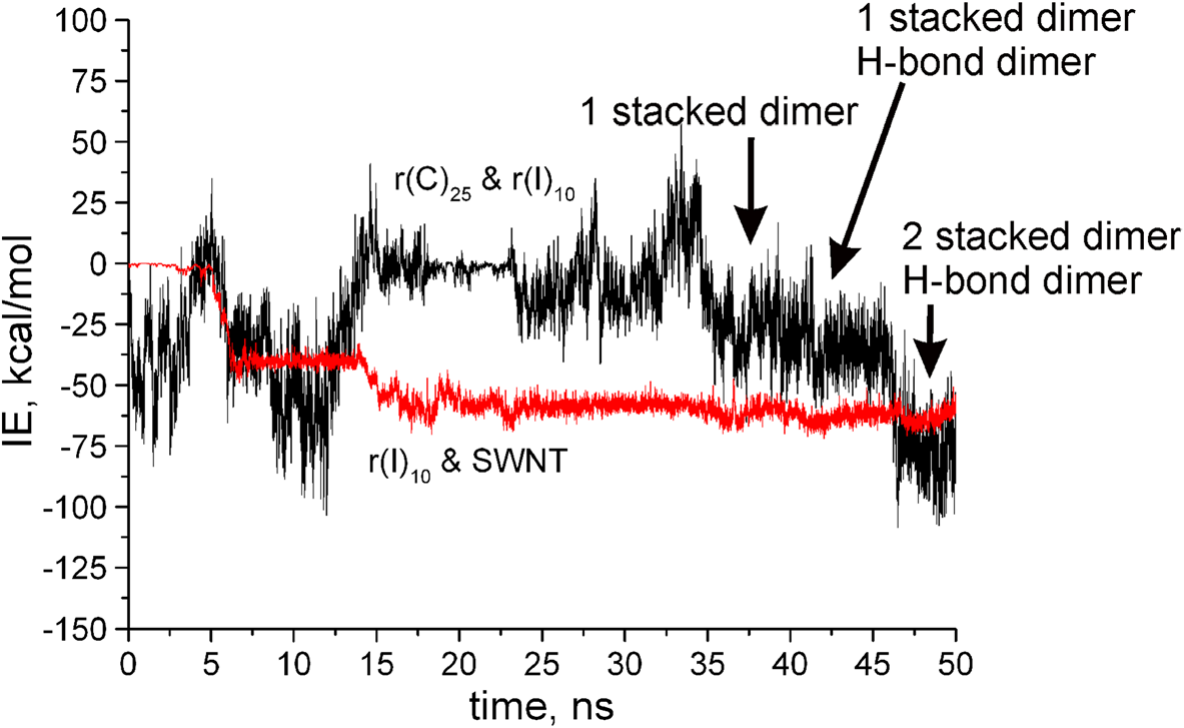
**Changes in the interaction energy.** Dependence of interaction energy between r(I)_10_ and r(C)_25_ adsorbed to SWNT (black), (rI)_10_ and SWNT (red) on simulation time at 343 K. Arrows indicate the appearance of stacked and H-bonded dimers.

After r(C)_25_ adsorption, the complementary oligomer r(I)_10_ was located near the hybrid prepared and then the system was modeled for the next 50 ns. To accelerate the hybridization process, r(I)_10_ was moved to r(C)_25_^NT^ from the side of one of its ends (Figure 4). The starting structure of r(I)_10_ was ordered in A-form. Upon simulation, this oligomer approaches the nanotube and interacts both with the nanotube surface and with r(C)_25_. The dynamics of interactions between components can be observed in Figure 5 which demonstrates changes in the interaction energy between different components of the system with time.

At first, we consider changes in the energy of interactions between r(I)_10_ and SWNT surface (Figure 5). A notable energy increment takes place after 5 ns of simulation when the oligomer approaches the nanotube and two or three bases (hypoxanthines) are adsorbed on its surface. At the same time, the binding energy of components of the complex reaches approximately 32 kcal/mol. The next energy growth (up to about 60 kcal/mol) takes place after 15 ns when the whole oligomer comes nearer to the nanotube, and this chain is placed practically transversely to the nanotube axis. However, the further simulation does not result in the increase of this energy value. It should be noted that r(I)_10_ oligomer moving along the tube is prevented by r(C)_25_ adsorbed earlier onto the nanotube, the conformation of which changes insignificantly with time.

Now we consider how the energy of the interaction between two oligomers depends on simulation time (Figure 5). First of all, we note the wide range of fluctuations in the interaction energy. Already at the beginning of simulation, the interaction energy reaches about 30 kcal/mol for a short time (<1 ns), and then the energy varies in the range of 10 to 30 kcal/mol with time. At 5 ns, the interaction between these oligomers weakens, approaching to the zero energy value, but then becomes stronger, mainly due to the appearance of the stacking dimer formed with the pair of cytosine-hypoxanthine. It should be noted that such a dimer is created several times and disrupted during modeling as heat vibrations of these two components exceed (or are close to) the value of the energy of their binding. This results in the absence of the interaction between oligomers in the 15- to 30-ns interval. Nevertheless, after 35 ns, the interaction between r(C)_25_^NT^ and r(I)_10_ begins to rise monotonically. First of all, cytosine-hypoxanthine stacking dimer is formed again, and at 44 ns, the cytosine-hypoxanthine flat dimer bound with two H-bonds is formed on the nanotube (Figure 5). Besides, at 50 ns, the stacking trimer hypoxanthine-cytosine-hypoxanthine is created, too (Figure 5). Note that these stacking complexes are formed at r(C)_25_^NT^ and r(I)_10_ ends, and this is readily explained as oligomer ends are more flexible. This mobility promotes the formation of the energetically favorable structures between two oligomers and facilitates the hybridization between them.

Thus, the hybridization process of two complementary oligomers on the nanotube surface occurs rather slowly, and we understand that the time scale taken is not enough to obtain complete statistics of this process. To observe the result of the hybridization, significant time (greatly more than 100 ns) is required. However, we believe that this time scale (up to 50 ns) is enough to describe at least the qualitative trend of the hybridization on the nanotube surface. This process is hindered with strong interaction of every oligomer with the nanotube surface. The polymer flexibility is necessary for quickly finding the most energetically favorable position between bases of two polymers, which results in the formation of H-bonded dimer.

From comparison of two processes (the base adsorption and hybridization) presented in Figure 5, it follows that the first one is more stable; after the base adsorption on the tube surface, the base desorption does not occur practically. While the hybridization is characterized by unstability of formed dimers which dissociate lightly and to stabilize this process, additional conditions (e.g., cooperativity or an additional interaction) are necessary. Besides, the formation of stacking structures of H-bonded dimers is hindered by the nanotube surface. In the free duplex, the stacking interaction stabilizes the new H-bonded dimer strongly and prevents its following decomposition, and this, in its turn, strengthens the double strand. To organize such stacking structures, the plane of H-bonded dimer must detach from the nanotube surface. But this step is prevented with strong π-π stacking interaction of bases with the nanotube surface. Besides, the curved nanotube surface distorts the plane of the dimer formed, and this weakens the H-bonded energy of the dimer. Thus, based on the above factors, it can be concluded that hybridization of two complementary oligomers on the nanotube is complicated because of their strong interaction with the nanotube surface. Formed on the curved nanotube surface, the H-bonded dimer is of weaker binding energy than the dimer created under usual conditions without surface.

## Conclusion

Hybridization of poly(rC) which is adsorbed to the carbon nanotube surface and free poly(rI) is hampered because of the strong surface-polymer interaction. Poly(rI) hybridization with poly(rC)^NT^ is characterized with a slow kinetics, the behavior of which differs essentially from hybridization of free polymers. The formation of double-stranded poly(rI)∙poly(rC)^NT^ is confirmed with the appearance of the S-like form of its melting curve representing the temperature dependence of the intensity of UV absorption. But parameters of this dependence differ substantially from those of free poly(rI)∙poly(rC): the melting temperature is decreased by 14°C, and the temperature range of helix → coil transition became wider essentially, starting practically from room temperature. In addition to it, the duplex on the nanotube is characterized with a lower hyperchromic coefficient. All these results indicate that the hybridization of two complementary homopolynucleotides occurs with deviation from the regular structure which is characterized by Watson-Crick pairing of bases. The spectral observation of defective hybridization on the carbon nanotube surface conformed to the results of computer simulation of this process. It was revealed that the strong interaction of nitrogen bases with the nanotube surface significantly weakens hybridization of two complementary oligomers, as the surface prevents the necessary conformational changes of the polymer to be hybridized. Also, computer simulation showed that before the nitrogen bases of two strands begin to form dimers (H-bonded or stacked ones), the free oligomer is adsorbed effectively to the nanotube surface, while dimers formed with bases of two strands are unstable and characterized with the hybridization/dissociation process.

The modeling results and their following discussion allow us to conclude that, upon the genosensor development employing nanotubes, the direct polymer adsorption onto the nanotube surface should be avoided.

## Competing interests

The authors declare that they have no competing interests.

## Authors’ contributions

MVK, GOG, and VAK conceived the present study. VSL prepared the samples. GOG performed the spectroscopic experiments. MVK and GOG processed the experimental data. MVK carried out the molecular dynamics simulation and analysis. VAK supervised the project. All authors contributed significantly to the discussions and to the manuscript writing. All authors read and approved the final manuscript.
